# Successful implementation of ITN distribution through health facilities in Ghana

**DOI:** 10.1186/s12936-023-04592-5

**Published:** 2023-08-02

**Authors:** Luigi Nuñez, Malia Skjefte, Obed E. Asamoah, Prince Owusu, Keziah Malm, Jane E. Miller

**Affiliations:** 1 PMI Vector Link, PSI, Washington DC, USA; 2 PMI Vector Link, PSI, Accra, Ghana; 3 National Malaria Elimination Programme, Accra, Ghana

**Keywords:** Malaria, ITN, ANC, EPI, CWC, Routine distribution, Continuous distribution, Pregnant women, Children, Ghana

## Abstract

**Background:**

Global efforts to reduce malaria burden include distribution of insecticide-treated mosquito nets through mass campaigns and routine channels. Ghana’s National Malaria Elimination Programme (NMEP) distributes insecticide-treated bed nets (ITNs) through various channels, including to pregnant women at antenatal care (ANC) visits and children at vaccination visits through child welfare clinics (CWC). This study assessed historical ITN distribution throughout ANCs and CWCs across Ghana and the characteristics of high performing facilities.

**Methods:**

Monthly data on routine ITN distribution was provided from Ghana’s national health information management system for the years 2016–2021. Analyses were conducted to assess the performance of ITN distribution at ANC and CWC across time, ecological zone, regions, districts, facility ownership, and facility type. Univariate and multivariate logistic regressions were performed to predict the odds of ANC and CWC issuing rates greater or equal to 80% for a given facility type or ownership.

**Results:**

In 2021, 93% of women who attended their first antenatal care visit and 92% of children under five who received their second dose of the measles-rubella vaccine (MR2) had received an ITN. At the regional level, 94% of regions (n = 15/16) maintained the NSP target issuing rate of 80% throughout 2020 and 2021. While there were no clear differences in issuing rates between ecological zones, district-level differences were present across the six years. All health facility types performed at or above 80% in 2021 for both ANC and CWC. Odds ratios demonstrated differences in the likelihood of meeting the 80% issuing rate goal among different facility types as well as private versus public ownership when comparing ANC and CWC.

**Conclusion:**

By 2021, Ghana had improved its ITN issuing rates since the initial year of analysis, surpassing the 80% target by issuing nets to over 90% of pregnant women and young children attending ANC and CWC. Future work can explore the reasons for national and subnational differences in issuing rates as well as help understand additional characteristics of high performing facilities. Additionally, it is necessary to identify and expand on the drivers for improved performance over the time period.

**Supplementary Information:**

The online version contains supplementary material available at 10.1186/s12936-023-04592-5.

## Background

Insecticide-treated bed nets (ITNs) have been a primary tool in the fight against malaria since the 1980s. In its 2021 World Malaria Report, the World Health Organization (WHO) estimated 1.7 billion malaria cases and 10.6 million malaria deaths were averted between 2000–2020 across the world, with 82% of these cases and 95% of the deaths averted from the WHO African Region [1]. An estimated 663 million cases of malaria were averted between 2000 and 2015, and an estimated 68% of these were due to ITNs [1]. Despite these gains, the global malaria community is concerned that these results are plateauing, as reductions in the rate of malaria cases have staggered. The recent COVID-19 pandemic has placed additional stress on health systems which has impacted malaria indicators [1]. About 85 countries were endemic with malaria in 2020, which together reported about 241 million cases of malaria, up from about 227 million malaria cases in 2019. Countries in the WHO African Region contributed most of this increase [1]. Malaria continues to be a major public health issue in Ghana. The country comprised 2.1% and 1.9% of global malaria cases and deaths, respectively [1]. According to Ghana’s 2016 Health Service Report, there were 10.4 million suspected malaria cases (38.7% of total outpatient cases seen), 4.5 million confirmed cases of malaria (16.8% of total outpatient cases seen), 379,986 malaria admissions (24.8% of total admissions), and 1,264 malaria deaths (4.2% of total deaths) in the country [2]. Furthermore, malaria continues to have a disproportionate impact on children in Ghana, as 46.7% of all malaria admissions and 46.7% of all malaria deaths were children under five years of age in 2016 [2].

Ghana started distributing nets in 1993, when a large-scale community-randomized trial of insecticide (Permethrin 50% EC) treated nets was conducted at Navrongo in Kassena-Nankana district, Upper East Region [3]. Since then, there have been many pilot interventions that ultimately resulted in the adoption of the current nationwide continuous distribution channels [4–10]. For example, in May 2004, the Department for International Development (DFID) and United States Agency for International Development (USAID) supported a pilot in Volta Region in which vouchers were given to pregnant women at their first antenatal visit that, when redeemed, provided a discount (if they could afford the top-up cash required) for a net purchased at private commercial retail outlets [5, 6]. In 2008, DFID provided 350,000 nets in Western Region to be used for routine distribution [7, 8]. These initiatives were based on a mixed model approach to ITN distribution, which subsidized distribution through the public and private sector, workplace and non-governmental organization distributions, full-cost sales, and occasional free distribution “catch-up” campaigns. In 2009, upon realizing that these approaches were not reaching the strategic targets nor certain populations (e.g., older children, adults) who contribute to malaria transmission, the NMEP shifted its policy towards “universal coverage” primarily by expanding mass distribution campaigns of ITNs for free [9]. Furthermore, in November 2011, the NMEP used NetCALC, an open-access population-based modelling tool, to select the combination of channels with estimates based on targets set at maintaining 90% of households owning at least one ITN [10]. A major component of this strategy was routine ITN distribution to pregnant women through ANC and infants through measles II booster visits at CWC.

There has been progress in reaching universal coverage of ITNs in the WHO African Region, especially in Ghana. Ghana’s 2019 Malaria Indicator Survey (MIS) found that 67% of residents had access to an ITN [11]. However, although more than half of the sampled residents had access, only 43% reported using the ITN the night before the survey [11]. Additionally, the Ghana 2019 MIS demonstrated clear differences in ITN population access and use among regions (e.g., 51% access in Greater Accra region vs 76% in Volta region), residence (e.g., 59% access among urban vs 74% among rural), and wealth quintiles (e.g., 71% access in the lowest wealth quintile vs 58% in the highest wealth quintile) [11]. With this said, the country is making great strides in acquiring and delivering nets. In 2020–2021, 16.7 million nets were delivered to Ghana by manufacturers [1]. This included 5.1 million pyrethroid-piperonyl butoxide (PBO) and 1.6 million dual active ingredient nets. Districts with the highest malaria burden and pyrethroid resistance were selected for the distribution of PBOs and the IG2 nets based on a national stratification.

Global efforts to reduce the burden of malaria include large-scale distribution of ITNs through mass campaigns and continuous distribution channels, among other efforts. CD channels include the routine distribution where nets are given to pregnant women in antenatal care (ANC) clinics and to young children during Expanded Programme for Immunization (EPI) visits, as well as schools, community-based distribution, and the private sector which includes social marketing. Using Demographic Health Surveys (DHS) from 25 African countries, a review by Theiss-Nyland et al*.* [12] found that an average of 54% of children slept under an ITN with both ANC and EPI distribution compared to 34% with ANC only and 24% with no facility-based distribution. These results highlight multiple advantages to having these channels operating efficiently. According to the World Malaria Report 2021, in Africa, 32 countries distributed ITNs through ANC and 24 through EPI clinics in 2020 [1].

One objective of Ghana’s strategy towards malaria control is to protect at least 80% of the population at risk with effective malaria prevention interventions by 2025 [13]. In Ghana, ITNs are issued to pregnant women on first contact at the ANC visit, and to children under five years of age during routine EPI at the second measle-rubella vaccine. The objective of these channels is to increase household and population access to ITNs by targeting pregnant women and children under five years, the most vulnerable groups to malaria.

The overall performance of the ANC and CWC channels has not been reviewed in detail since Ghana began implementation nor have the nuances across varying levels (including region, district, ecological zones, facility types, and ownership) been explored. Even at the global level, there is little public information. For example, a recent multi-country analysis found that the national ITN issuing rates at ANC across seven sub-Saharan countries and EPI for six sub-Saharan countries were 65% and 69%, respectively [14]. To help fill this knowledge gap, a historical analysis of bed net issuing rates through ANCs and CWCs across six years (2016–2021) was conducted. These study results highlight national and subnational trends in issuing rates across the country and provide critical information and insights to influence future decision-making and resource allocation on the topic of malaria prevention. Additionally, results can be used to guide the creation or revision of impactful distribution channels in other high-burden countries.

## Methods

### Study design and data collection

A retrospective, longitudinal study design was chosen to identify and analyze collected data from 2016–2021. In Ghana, health facilities collect routine data as part of service delivery using standard registers, tally sheets and stock inventory control cards. The health facilities then collate the data from these source documents into standardized reporting forms. Data from these forms are then entered into the electronic software called District Health Information Management System (DHIMS), which is Ghana’s central system based on District Health Information Software (DHIS2), an open-source software platform for reporting, analysis, and dissemination of data used in more than 60 countries around the world. The Ghana Health Service, based on the Standard Operating Procedures for Health Information Management, expects all facilities to complete data compilation and entry by the fifth day of the ensuing month. Facilities/sub-districts without data entry capacity must submit their data to the district to be entered into DHIMS. For this case-study, ITN distribution data was retrieved from the Monthly Midwife’s Form A for the ANC and the EPI Monthly Vaccination Report for the CWC.

### Data reporting

Health facilities have up to the fifth day of the following month to enter the data directly into DHIMS or submit their data if they do not have data entry capacity. The district has ten days to verify, validate and enter data into DHIMS before signing off electronically on the 15th of the same month. The Regional Health Directorate also has ten days to review the data, check for internal consistency, and sign off electronically on the 25th of the ensuing month. Health facilities have a period of 60 days within which they can update data submitted to the next level. After 60 days, the data is locked, and no further amendments can be made unless the facility fills an amendment form endorsed by the regional health directorate. Between 2016 and 2021, the reporting rate, pulled from DHIMS, ranged between 91.7% and 97.0% for the ANC Monthly Form A and between 97.0% and 100.0% for the Monthly Vaccination Report.

### Data verification and validation

As part of the Standard Operating Procedures for Health Information Management, each facility has a data validation team to verify and validate data reported to the national database. At the end of the month, each facility head in Ghana reviews and endorses collated facility/sub-district data after the facility data validation teams have approved it and before it is forwarded to the district level. At the district level, data validation teams also look at the internal consistency of data by comparing related indicators (e.g., the number of ANC registrants should be equal to the sum of age groups for Age of mother at registration within the ANC Monthly Form A). In addition to these routine checks, the NMEP and partners also carry out data quality checks. For example, the ITN vector control team has instituted a quarterly desk data review and feedback at the NMEP. During this activity, the team reviews the performance of the various regions, districts, and facilities while checking for the internal consistency of data. In addition, the national team provides feedback to the regional malaria focal persons who follow up on issues identified by the team and ensures they are corrected.

### Data analysis

The performance of CD of ITNs through health facilities is measured through the issuing rate at ANC and CWC.

The issuing rate at ANC is calculated as the number of pregnant women who received an ITN at ANC divided by the number of pregnant women at their first ANC visit times 100%.

$${Issuing\; rate}_{ANC}=\frac{\#\; of\; pregnant\; women\; who\; received\; an\; ITN\; at\; ANC}{\#\; of\; pregnant\; women\; at\; first\; ANC\; visit} x 100\%$$

The issuing rate at CWC is calculated as the number of children who received an ITN at CWC divided by the number of children receiving their second vaccine dose for measles-rubella times 100%.

$${Issuing\; rate}_{CWC}=\frac{\#\; of\; children\; who\; received\; an\; ITN\; at\; CWC}{\#\; of\; children\; receiving\; MR2\; vaccine\; dose\; at\; CWC} x 100\%$$

These four elements are reported by health facilities as part of monthly routine reporting of health services to the government of Ghana. As previously mentioned, these data are entered into the national DHIMS which can then be analysed through live dashboards or exported for further analyses in other software such as Excel or STATA.

**Fig. 1:**
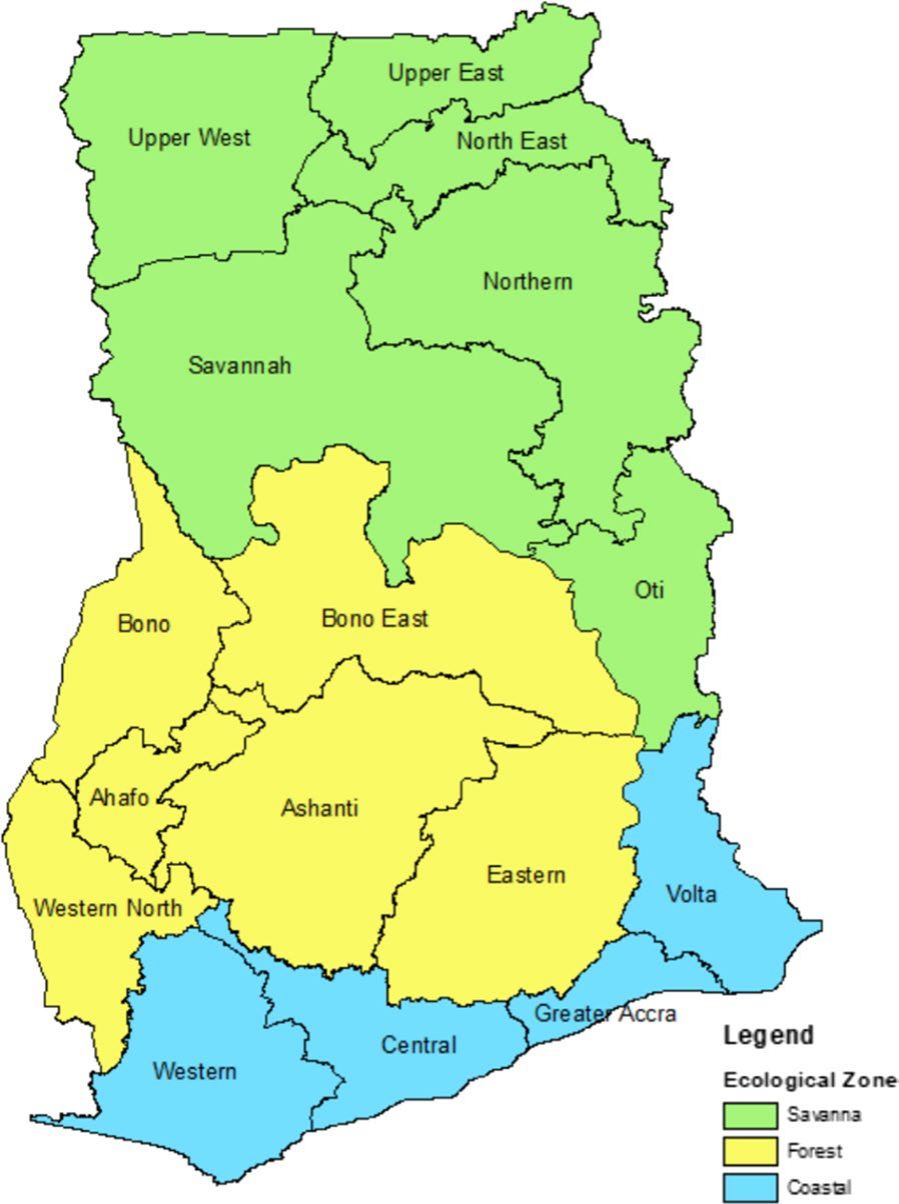
Administrative regions of Ghana and their ecological designation

Using data at the facility level, analyses were conducted to visualize trends in ITN issuing rates over time as well as determine any characteristics that potentially influence issuing rate. The facility list used in the analyses was exported from DHIMS and disaggregated by region, district, facility type, facility ownership, and ecological zone. Definitions for each facility type and facility ownership can be found in Tables 1 and 2, respectively. Regions were assigned one of three ecological zones (Forest, Coastal, or Savanna) based on groupings from the National Malaria Strategic Plan 2021–2025 (Fig. 1).

**Table 1:** Definitions for type of health facility

Health Facility Type	Definition
CHPS	Community Based Health Planning and Services (CHPS) is a national mechanism to deliver essential community-based health services involving planning and service delivery with the communities. It is the first point of call. Its primary focus is communities in deprived subdistricts and, in general, bringing health services close to the community. It is a clearly defined area within a subdistrict where a community health officer provides community-based health services, including home visits to clients residing in the CHPS zone. Some CHPS compounds also have a midwife attached to provide reproductive health services
Clinic	Clinics do not provide a full range of services. They usually provide Reproductive and Child Health or basic curative services. It is designed to provide only curative care and screening for referrals to the health centre/ district hospital. Facilities of this type are mostly found at the community level, and most are private
Health Centre	Health centres provide a full range of basic primary healthcare services (clinical, public health, and maternity services). It is usually staffed by a physician assistant, midwife/midwives, enrolled nurses, and community health officers. Facilities of this type are mostly found at the sub-district level
Polyclinic	Polyclinics are usually sited in urban/ cosmopolitan areas to ease the congestion in teaching and regional hospitals and serve as large health centres to ensure that primary care services are evenly distributed and provide a range of services that are higher than a health centre. Polyclinics are planned with excess capacity for future expansion and development as district hospitals. These facilities provide 24-h services
Hospital	A hospital comprises of a wide range of services and functional units. These include diagnostic and treatment functions, such as clinical laboratories, imaging, emergency rooms, and surgery; hospitality functions, such as food service and housekeeping; and the fundamental inpatient care or bed-related function District hospital: District hospitals serve geographically defined areas and are considered first referral facilities, providing a range of clinical services, including emergency services, inpatient care, laboratory testing, and surgeries. District hospitals provide a full range of primary healthcare services of a general hospital under the management of at least two general duty doctors Regional hospital: Regional hospitals that provide more specialized care and the next level of referral for more complicated cases, in addition to general inpatient care, outpatient services, laboratory care, and surgeries
Maternity Home	Maternity homes are health facilities that focus on providing reproductive and family planning services
Medical Centre	Medical centres are private/quasi-governmental facilities that operate like district/specialized hospitals
Other	Refers to facilities that do not clearly fall within the categorizations above. Select examples include research institutions, Seventh Day Adventist facilities, and medical reception stations

**Table 2:** Definitions for ownership of health facility

Health Facility Ownership	Definition
Private	Private health facilities include (1) private facilities (i.e., health facilities owned by private individuals or institutions); (2) Christian Health Association of Ghana (CHAG), a network of health facilities owned by 21 different Christian church denominations that provide health care to the most vulnerable and underprivileged population groups in all 16 regions of Ghana, particularly in the most remote areas; (3) other faith-based (i.e., health facilities owned by other non-Christian religious organizations such as the Ahmadiyya Muslim hospitals); and (4) mines (i.e., health facilities owned by mining companies)
Public	Public health facilities include (1) government facilities (i.e., health facilities that are government-owned and funded by the government) and (2) quasi-government (i.e., health institutions that comprise government institutions, agencies, and departments whose primary service focuses not on health but engaging in health service provision, such as police hospitals, military hospitals, and Ghana atomic energy hospitals)

Key tables and graphs were created using Microsoft Excel 365 to visualize patterns and trends in ITN issuing rates over the course of six years (2016–2021). Change variables were calculated to see the overall improvement in issuing rates from initial year (2016) to the final year (2021).

ITN issuing rates were also mapped at the district level with a regional overlay for ANC and CWC for each year to see if districts were reaching the target goal of 80%. Additional maps were created to understand similarities and differences between ANC and CWC issuing rates by year. All maps were created using ArcGIS Desktop 10.8.2.

STATA 17 was used to perform univariate and multivariate logistic regressions. These regressions were conducted to predict the odds and adjusted odds of ANC and CWC issuing rates greater or equal to 80% for a given facility type or facility ownership. For these regressions, only 2021 data was used to gain insight on current relationships between issuing rates and a given characteristic. Health centre was chosen as the reference variable for the health facility calculation while private was chosen as the reference variable for the ownership calculation.

## Results

The analyses considered 9,638 health facilities as per DHIMS (Table 3). Almost half (44.8%) were in Ashanti, Eastern, and Greater Accra regions; most (71.9%) were CHPS; most (85.4%) were government-run; and about half (51.4%) were in the forest ecological zone.

**Table 3:** Demographics of DHIMS data

	Number of health facilities	Percent of total (%)
Region		
Ahafo	176	1.8
Ashanti	2575	26.7
Bono	418	4.3
Bono East	353	3.7
Central	626	6.5
Eastern	1106	11.5
Greater Accra	1059	11.0
North East	131	1.4
Northern	446	4.6
Oti	238	2.5
Savannah	180	1.9
Upper East	531	5.5
Upper West	422	4.4
Volta	489	5.1
Western	558	5.8
Western North	330	3.4
Total	9638	100.0
Type of health facility		
CHPS	6926	71.9
Clinic	569	5.9
Health Centre	1094	11.4
Hospital	536	5.6
Maternity Home	236	2.4
Medical Centre	97	1.0
Other	109	1.1
Polyclinic	71	0.7
Total	9638	100.0
Ownership of health facility		
Public	8235	85.4
Private	1403	14.6
Total	9638	100.0
Ecological zone		
Coastal	2732	28.3
Forest	4957	51.4
Savanna	1949	20.2
Total	9638	100.0

Type of health facility + Ownership of health facility	Private (% of facility type total)	Public (% of facility type total)	Total
CHPS	195 (2.8%)	6731 (97.2%)	6926
Clinic	415 (72.9%)	154 (27.1%)	569
Health centre	97 (8.9%)	997 (91.1%)	1094
Hospital	325 (60.6%)	211 (39.4%)	536
Maternity home	217 (91.9%)	19 (8.1%)	236
Medical centre	91 (93.8%)	6 (6.2%)	97
Other	56 (51.4%)	53 (48.6%)	109
Polyclinic	7 (9.9%)	64 (90.1%)	71
Total	1403	8235	9638

All 16 regions started under 80% issuing rate in 2016 at ANC, and only two (Ahafo and Bono East) performed above 80% in 2016 at CWC. All regions performed above target in 2020 and 2021, except for Western North at ANC in both 2020 and 2021. Almost all pregnant women at first ANC (93%) and children under five receiving MR2 (93%) received an ITN in 2021 (Table 4). Nationally this is an improvement of 53 percentage points at ANC and 39 percentage points at CWC since 2016. Additionally, when comparing overall change between ANC and CWC, it is evident that a greater net change took place at ANCs versus CWCs over time, with CWC rates starting higher than ANC in the initial years (Table 4).

**Table 4:** Total issuing rate at ANC and CWC by region

Total Issuing Rate	2016		2017		2018		2019		2020		2021		Change 2016–2021	
Region	ANC (%)	CWC (%)	ANC (%)	CWC (%)	ANC (%)	CWC (%)	ANC (%)	CWC (%)	ANC (%)	CWC (%)	ANC (%)	CWC (%)	ANC (%)	CWC (%)
Ahafo	72	83	97	99	96	94	94	95	98	97	86	95	13 ▲	13 ▲
Ashanti	29	39	55	64	83	84	83	77	95	86	97	93	68 ▲	55 ▲
Bono	64	70	97	97	100	98	93	91	97	95	100	98	36 ▲	28 ▲
Bono East	64	87	96	99	95	97	78	84	95	96	85	90	21 ▲	3 ▲
Central	20	28	62	63	80	76	91	90	88	85	101	98	80 ▲	69 ▲
Eastern	48	60	93	96	99	98	98	97	98	97	97	95	49 ▲	35 ▲
Greater Accra	32	46	75	84	58	53	71	64	88	88	91	89	59 ▲	43 ▲
North East	47	65	65	61	56	56	83	81	86	90	92	94	45 ▲	29 ▲
Northern	43	65	71	72	67	66	56	56	90	84	84	88	41 ▲	22 ▲
Oti	30	44	88	93	98	98	95	97	94	96	99	96	69 ▲	52 ▲
Savannah	55	69	81	80	87	84	93	88	92	85	92	85	37 ▲	16 ▲
Upper East	72	78	76	80	89	83	83	85	96	96	105	94	33 ▲	17 ▲
Upper West	12	29	86	95	86	86	89	93	96	94	96	99	84 ▲	70 ▲
Volta	44	61	95	96	99	99	98	99	98	96	99	98	55 ▲	37 ▲
Western	51	64	92	93	98	91	97	93	92	82	86	82	35 ▲	18 ▲
Western North	52	59	95	96	94	91	97	95	71	80	74	87	23 ▲	27 ▲
Grand Total	40	53	77	82	82	81	84	82	92	89	93	92	53 ▲	39 ▲

▲Indicates a net increase in issuing rate

**Fig. 2:**
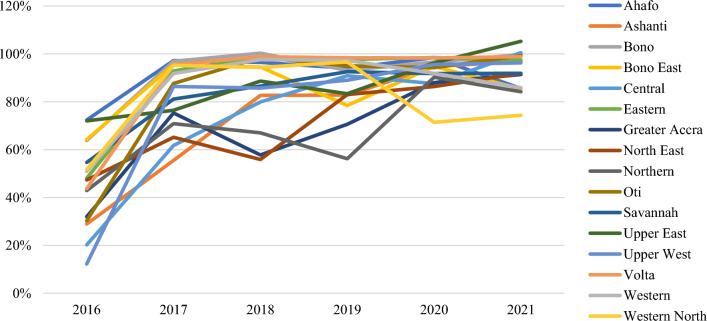
Annual issuing rate (ANC) by region

**Fig. 3:**
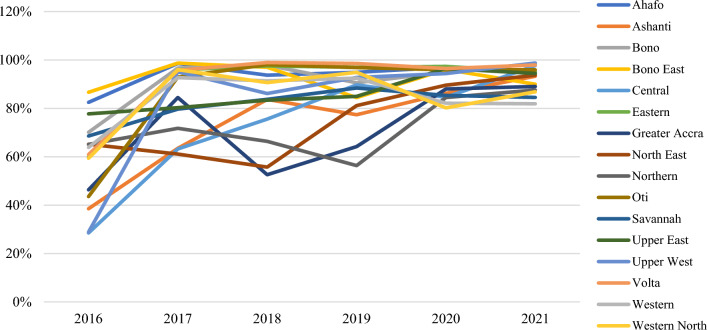
Annual issuing rate (CWC) by region

In general, monthly performance consistently improved for all regions at both ANC and CWC (Additional file 1: Figs. S1, S2, Figs. 2, 3). At ANC, regions performed at worst 12% in 2016 and at worst 74% in 2021. At CWC, regions performed at worst 28% in 2016 and at worst 82% in 2021. Ashanti, Central, Oti, and Upper West regions all experienced a greater than 50% increase in issuing rate when comparing the final and initial years. There were a handful of monthly anomalies to note. For ANC, the Northern and Upper East regions had an 185% issuing rate in December 2020 and September 2021, respectively. For CWC, both the Bono and Ahafo regions had spikes above 140% in July 2017. Assuming data are validated and confirmed accurate, it is still possible for issuing rates to be above 100%. For example, pregnant women who did not receive an ITN at their first visit are still eligible to receive a net at a subsequent visit, children who did not receive an ITN at the MR2 vaccination visit are still eligible to receive a net at a subsequent visit, and children who reach the age for MR2 are still eligible for an ITN even if there is a stockout of the MR vaccine at the facility. Overall, Ghana has improved its ITN issuing rates, surpassing the 80% target by issuing nets to over 90% of pregnant women and young children attending ANC and CWC clinics.

**Fig. 4:**
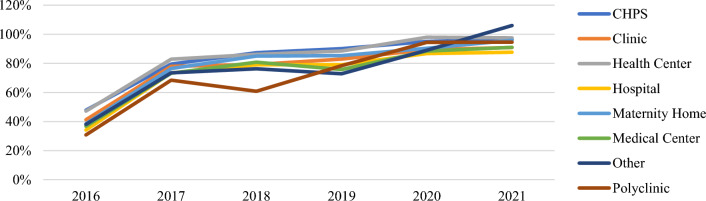
Annual issuing rate (ANC) by facility type

**Fig. 5:**
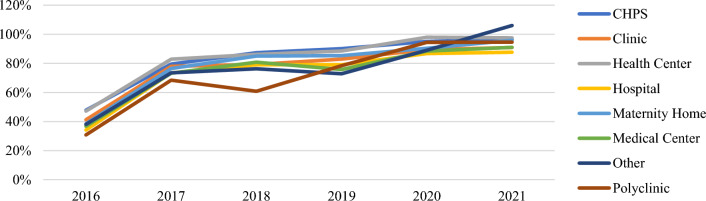
Annual issuing rate (CWC) by facility type

In addition, performance by type of facility also improved between 2016 and 2021 (Additional file 1: Figs. S3, S4, Table 5). While all types of health facilities performed under 80% in 2016 for both ANC and CWC, all performed above 80% in 2021. All even performed above 90% at both ANC and CWC except for clinics and maternity homes at CWC (88% and 89%) and hospitals at ANC (88%). Monthly performance also continuously improved for all types of health facilities at both ANC and CWC between 2016–2021. “Other” facility types saw the largest net change from 2016 to 2021 with a 68% increase (Figs. 4, 5). All facility types met the 80% target in 2021.

**Table 5:** Total issuing rate at ANC and CWC by health facility type

Total Issuing Rate	2016		2017		2018		2019		2020		2021		Change 2016–2021	
Facility	ANC (%)	CWC (%)	ANC (%)	CWC (%)	ANC (%)	CWC (%)	ANC (%)	CWC (%)	ANC (%)	CWC (%)	ANC (%)	CWC (%)	ANC (%)	CWC (%)
CHPS	48	54	80	81	87	83	90	83	95	90	97	92	49 **▲**	38 **▲**
Clinic	41	51	78	81	79	75	83	77	89	86	91	88	50 **▲**	37 **▲**
Health Centre	47	55	83	83	86	83	89	85	98	91	98	95	50 **▲**	40 **▲**
Hospital	34	54	73	84	79	72	79	73	87	85	88	90	54 **▲**	37 **▲**
Maternity Home	38	44	76	76	85	74	85	73	90	81	96	89	58 **▲**	45 **▲**
Medical Centre	37	48	73	70	81	70	76	65	88	88	91	93	54 **▲**	45 **▲**
Other	38	49	73	82	76	60	73	69	89	94	106	95	68 **▲**	47 **▲**
Polyclinic	31	42	68	73	61	70	78	79	95	92	95	91	64 **▲**	49 **▲**

▲Indicates a net increase in issuing rate

**Fig. 6:**
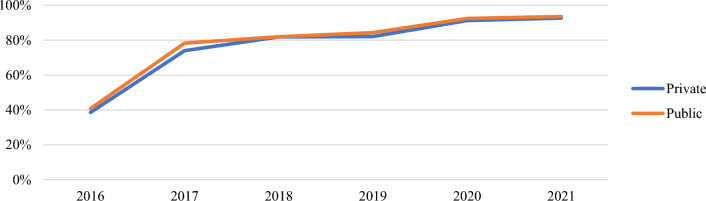
Annual issuing rate (ANC) by ownership

**Fig. 7:**
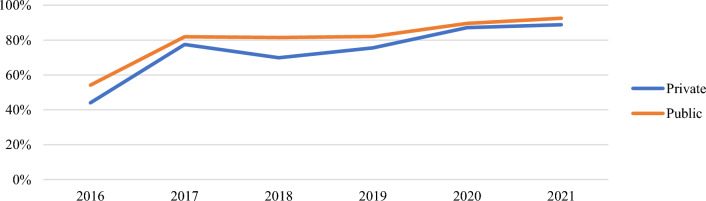
Annual issuing rate (CWC) by ownership

Performance by ownership also varied at both ANC and CWC (Additional file 1: Figs. S5, S6, Table 6). Public ownership included facilities owned by the government and quasi-government while private ownership included facilities owned by CHPS, other faith-based organizations, mines, and private organizations. While neither public nor private ownership types met the 80% target in 2016, both ownership types hit the mark at ANC and CWC by 2021 (Figs. 6, 7). Both ownership types experienced the largest increase in total issuing rates between 2016 to 2017. Overall, both private and publicly owned facilities experienced a roughly 30% increase in ITN distribution since 2016.

**Table 6:** Total issuing rate at ANC and CWC by health facility ownership

Total Issuing Rate	2016		2017		2018		2019		2020		2021		Change 2016–2021	
Ownership	ANC (%)	CWC (%)	ANC (%)	CWC (%)	ANC (%)	CWC (%)	ANC (%)	CWC (%)	ANC (%)	CWC (%)	ANC (%)	CWC (%)	ANC (%)	CWC (%)
Private	39	44	74	77	82	70	82	76	91	87	93	89	38 **▲**	30 **▲**
Public	41	54	78	82	82	81	84	82	92	90	94	93	38 **▲**	27 **▲**

▲Indicates a net increase in issuing rate

**Fig. 8:**
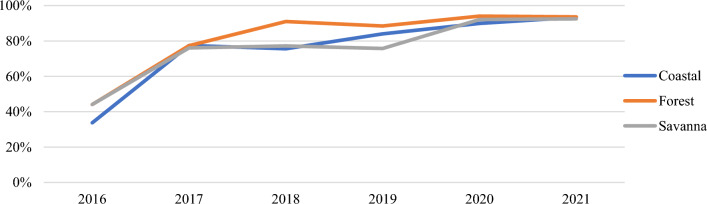
Annual issuing rate (ANC) by ecological zone

**Fig. 9:**
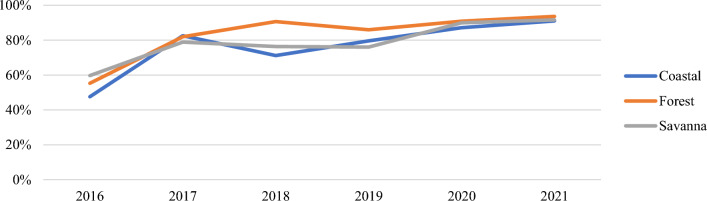
Annual issuing rate (CWC) by ecological zone

Trends in issuing rates were similar across the three ecological zones (Coastal, Forest, and Savanna) over the six years with a large increase occurring from 2016 to 2017 (Additional file 1: Figs. S7, S8, Table 7). Of note, there was a spike in November and December in the Savanna ecological zone for ANC and CWC (Figs. 8 and 9).

**Table 7:** Total issuing rate at ANC and CWC by ecological zone

Total Issuing Rate	2016		2017		2018		2019		2020		2021		Change 2016–2021	
Ecological Zone	ANC (%)	CWC (%)	ANC (%)	CWC (%)	ANC (%)	CWC (%)	ANC (%)	CWC (%)	ANC (%)	CWC (%)	ANC (%)	CWC (%)	ANC (%)	CWC (%)
Coastal	34	48	77	83	76	71	84	80	90	87	93	91	60 **▲**	43 **▲**
Forest	44	55	77	82	91	91	89	86	94	91	94	94	50 **▲**	38 **▲**
Savanna	44	60	76	79	77	76	76	76	92	90	93	92	33 **▲**	32 **▲**

▲Indicates a net increase in issuing rate

**Fig. 10:**
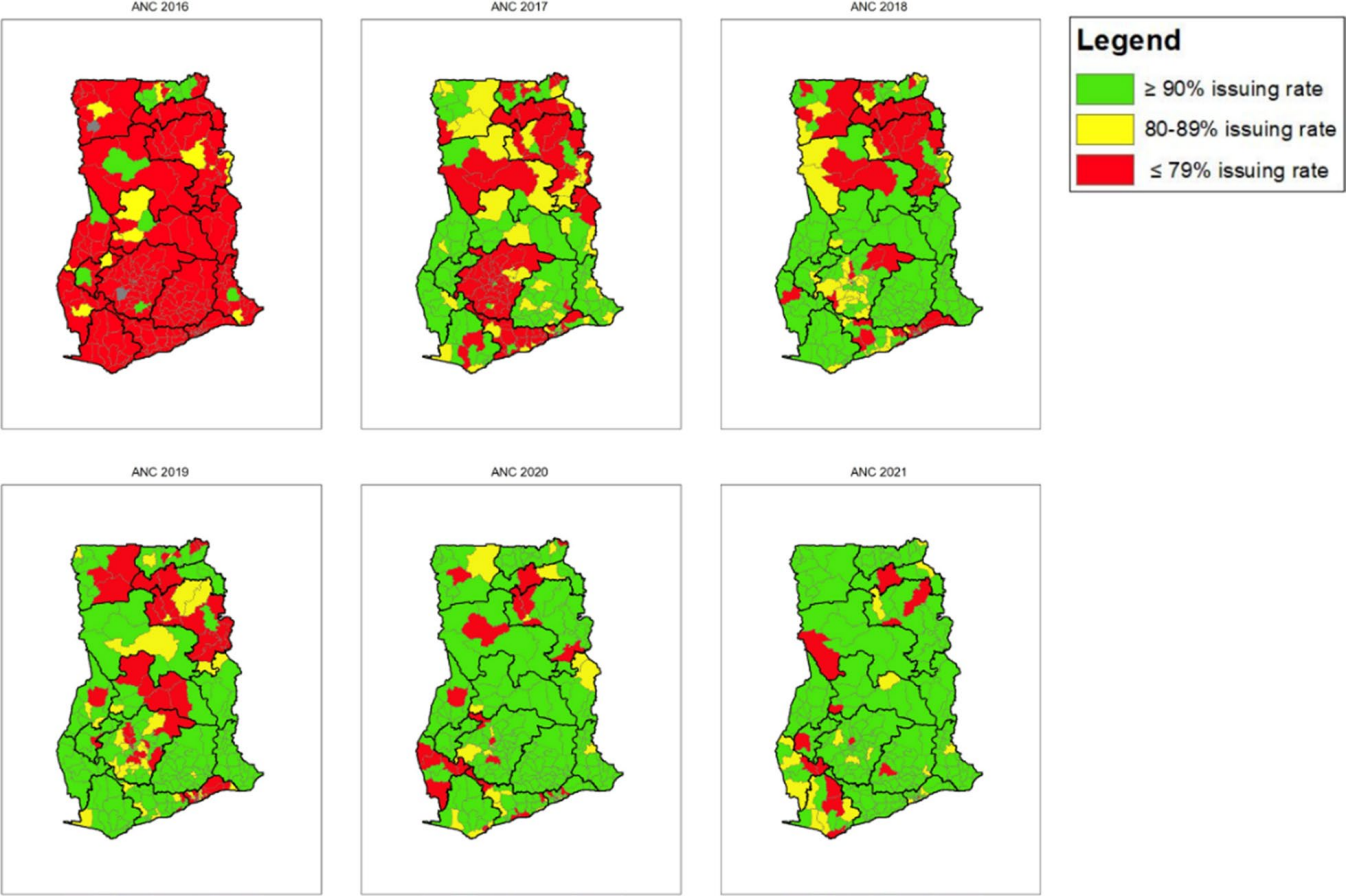
ANC ITN issuing rate per district map (2016–2021)

**Fig. 11:**
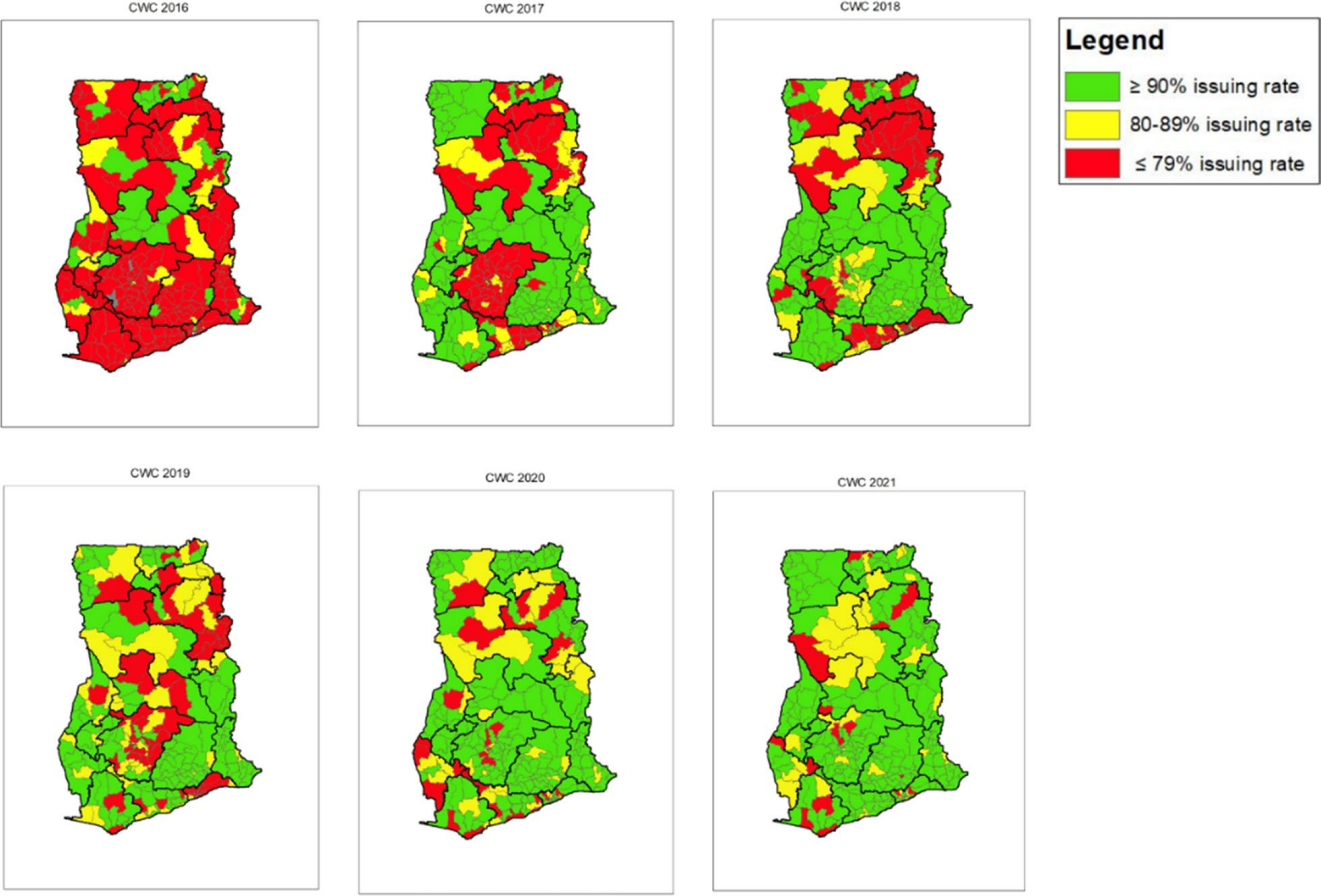
CWC ITN issuing rate per district map (2016–2021)

Improvements in ITN issuing rates over time can also be seen at the district level for both ANC and CWC. Figures 10 and 11 show a geographical visual of the annual ANC and CWC ITN issuing rates per district (Table 8) over six years, from 2016 to 2021. The largest increase in issuing rates for both ANC and CWC occurred between 2016 and 2017, where there were shifts from issuing rates under 79% to issuing rates at least 80 or 90% in some districts. By 2021, 92% of districts had reached an issuing rate of at least 80% at ANC and 90% of districts had reached an issuing rate of at least 80% at CWC. Furthermore, 85% of districts reached an issuing rate of at least 90% at ANC and 76% of districts reached an issuing rate of at least 90% at CWC. For both ANC and CWC, most districts in Bono, Bono East, Central, Eastern, Greater Accra, Oti, Upper West, and Volta regions reached the 80% target goal in 2021. Most districts that did not reach the 80% issuing rate goal by 2021 were located in Ashanti, Northern, North East, Savannah, Western, and Upper East regions. At ANC, the lowest performing district in 2016 had an issuing rate of 0% and the lowest performing in in 2021 had an issuing rate of 45%. At CWC, the lowest performing district in 2016 had an issuing rate of 0% and the lowest performing in 2021with an issuing rate of 47%.

**Fig. 12:**
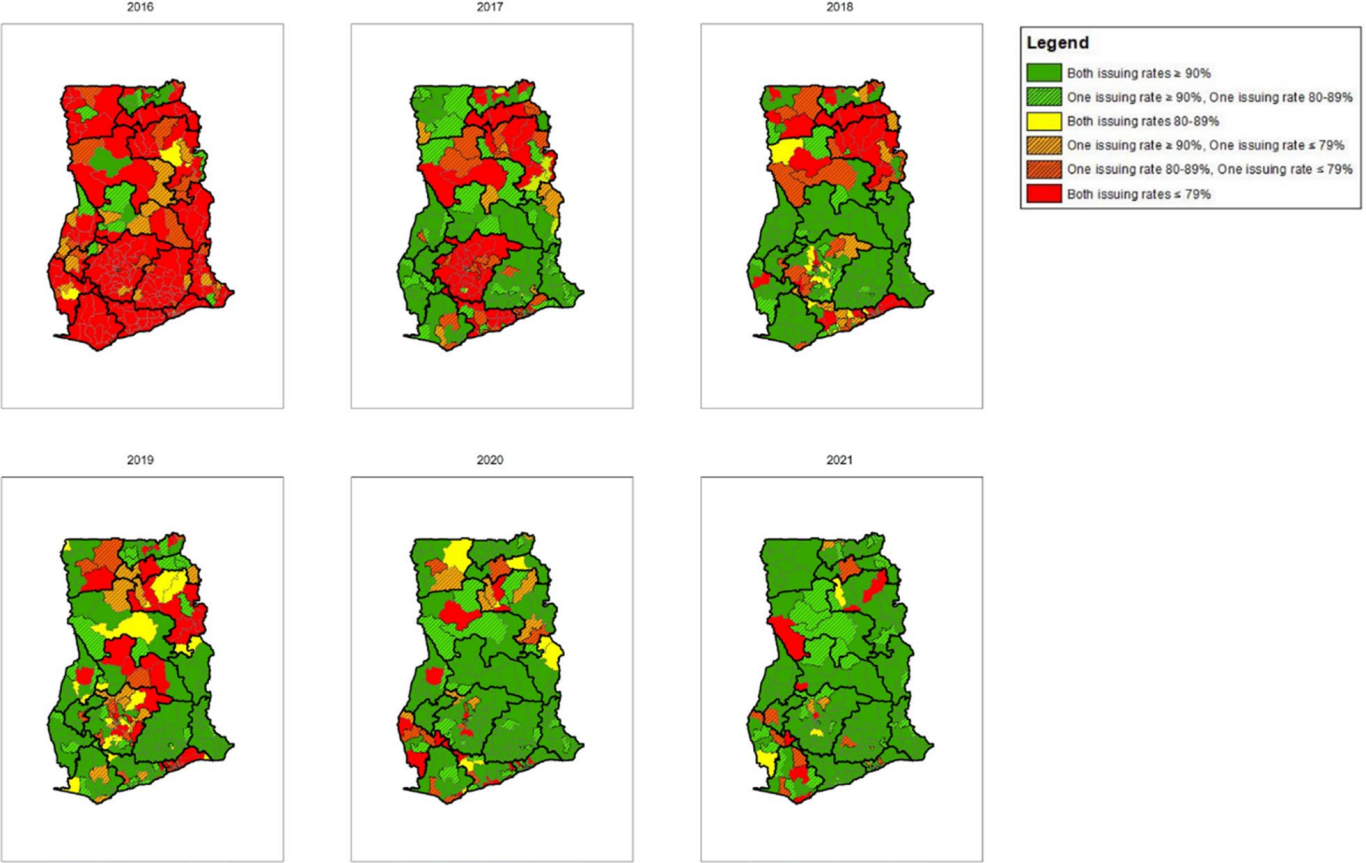
Combined ANC and CWC ITN issuing rates per district map (2016–2021)

Maps were also created to visualize the similarities and differences between ANC and CWC issuing rates for the same district for a given year (Fig. 12). These maps show that most districts had similar issuing rates between ANC and CWC, with few districts experiencing an issuing rate “mismatch”, where there was at least a 10-point difference in issuing rates between ANC and CWC for the same year. The districts that experienced the largest mismatch between ANC and CWC in 2021 were also located in the northeast and southwest regions of the country.

Odds ratios were used to predict the likelihood of an issuing rate greater or equal to 80% for a given characteristic of facility type or ownership given a 95% confidence interval. Compared to health centres, CHPS facility types were 2.28 times more likely to perform at least 80% at ANCs in 2021. On the other hand, CHPS CWCs were only 0.73 times more likely to have an issuing rate of at least 80% issuing rate in the same year than health centres. Polyclinics also had high odds of reaching an issuing rate of at least 80% issuing rate at ANCs (OR = 1.60) compared to CWCs (OR = 0.76). Interestingly, maternity homes had higher odds of meeting the 80% target at CWCs (OR = 1.30) versus ANCs (OR = 0.71) for 2021. Compared to private ownership, publicly owned ANCs were 4.56 times more likely to meet the 80% target in 2021 while publicly owned CWCs were only 0.84 times more likely to reach the goal. Moreover, privately owned ANCs were only 0.21 times more likely to perform at ≥ 80% compared to government owned ANCs.

**Table 8:** Univariate and multivariate logistic regression predicting ANC and CWC ITN issuing rates ≥ 80% among ownership and facility types

		ANC Issuing Rate ≥ 80%				CWC Issuing Rate ≥ 80%			
Characteristic		OR	P-Value	Adjusted OR	P-Value	OR	P-Value	Adjusted OR	P-Value
Facility Type (Ref: Health Centre)	CHPS	2.28	p < 0.001	2.20	p < 0.001	0.73	p < 0.001	0.74	p < 0.001
	Clinic	0.39	p < 0.001	0.54	p < 0.001	0.74	p < 0.001	0.63	p < 0.001
	Hospital	0.33	p < 0.001	0.44	p < 0.001	0.67	p < 0.001	0.58	p < 0.001
	Maternity Home	0.71	p = 0.001	1.09	p = 0.45	1.30	p < 0.01	1.03	p = 0.77
	Medical Centre	0.37	p < 0.001	0.56	p < 0.001	0.86	p = 0.19	0.69	p = 0.003
	Polyclinic	1.60	p = 0.03	1.60	p = 0.030	0.76	p = 0.020	0.76	p = 0.020
	Other	0.80	p = 0.13	1.02	p = 0.89	1.92	p < 0.001	1.71	p = 0.001
Ownership (Ref: Private)	Public	4.56	p < 0.001	1.60	p < 0.001	0.84	p < 0.001	0.79	p < 0.001

## Discussion

This activity aimed to showcase trends in ITN issuing rates at ANC and CWC in Ghana over six years and identify potential factors of high performance. Analyses were done using nationally aggregated results and were disaggregated by ecological zone, region, district, facility type, and facility ownership.

*Change overtime:* Overall, results show a clear and substantial increase in issuing rates at both ANC and CWC for all disaggregation levels throughout the six-year period. Ghana has exceeded national strategic targets for distribution of ITNs at health facilities and maintained high performance even throughout 2020 and 2021, a time of calamity that was the COVID-19 pandemic. It is possible these results stemmed from a variety of drivers, such as improved supply chains, strong leadership implementing a centralized approach, supportive policies and guidelines, improved data quality, and implementation of other activities that support facility operations and provider competency and adherence such as supportive supervision. However, this activity did not aim to answer this question.

*Variation across regions:* Logically, the majority of facilities were located in three regions (Ashanti, Eastern, and Greater Accra) since nearly 44.8% of Ghana's population live in these three regions [15]. These three regions also tended to be amongst the top performers between 2016 and 2021 (Ashanti, 68% increase at ANC and 55% increase at CWC; Eastern, 49% increase at ANC and 35% increase at CWC; Greater Accra, 59% increase at ANC and 43% at CWC). This may be due to unintentional increased focus on these regions due to the population catchments or other activities supporting facility operations that may have also benefitted facility-based ITN channels.

*Variation across districts:* The variation at the district level is even higher than that at regional level, which is likely representative of the larger health system differences including, but not limited to, access to healthcare services, ease of transport of commodities through rough terrain or bodies of water (noting also the typical large size of bales for ITNs), such as lakes and rivers, and the ability to get information about updated policies and guidelines to more secluded facilities (which also likely lack or have intermittent internet access and electricity).

*Variation across ecological zones.* Performance across ecological zones were similar. In general, all three zones grew, dipped, and peaked either at the same time or around similar time periods. All three zones performed between 91 and 94% in 2021, insinuating that the ecological zone of the country is not likely a factor to performance and resource allocation based on ecological zone may not benefit progress towards the malaria strategic plan.

*Predictors of meeting ITN strategic targets:* These analyses also provided insights into how facility types and ownership correlated with different results, particularly in meeting the 80% strategic target established in the most recent National Malaria Strategic Plan. CHPS and polyclinics were predicted to be more likely to meet the 80% strategic target at ANC (2.28 and 1.60 times, respectively) than health centres, while maternity homes were more likely to meet the 80% strategic target at CWC (1.30) than health centres. This analysis aligns with the predictions found under the ownership, since 97.2% of CHPS and 90.1% of polyclinics were public and 91.9% of maternity homes were private. Public health facilities were 4.56 times more likely to meet the 80% target at ANC than private health facilities while only 0.84 times as likely to meet the 80% target at CWC. Even after adjusting with the multivariate analyses, public health facilities were still 1.60 times more likely to meet the 80% target at ANC than private health facilities and 0.79 times at CWC.

More work needs to be done to understand predictors of meeting the 80% targets, as there may be various factors impeding optimal facility-based distribution [16]. It may be that stock availability and net cost affect facilities meeting the 80% targets, since different types of facilities receive commodities differently. Other literature has also reported how education level, knowledge of malaria, community involvement, socio-economic status, and party are main determinants to ownership of ITNs, and there may be some extension to how these determinants (in addition to others, such as low awareness of need, seasonal variations in use, and discomfort) affect facility-based distribution (e.g., not being aware of free ITNs at the facility, inability to return to a facility if there is a stock out during the visit, not wanting the ITN even if provided) [17]. Stock availability could also be an issue in facilities located in rural areas, and other literature have found performance indicators for facility-based healthcare services performing better in urban facilities than those in rural areas. Private health facilities that do not receive commodities from the regional medical stores are less likely to request ITNs if district health teams do not facilitate the requisition process, so it may be that private health facilities experience stockout more often than government-owned facilities. For example, a 2021 study mapping ITN access and use in Africa shared that optimal ITN performance is challenged by distribution, utilization, and retention, in which the African continent faces supply-chain and commodity challenges making it especially difficult for sufficient net acquisition at the household level [18].

## Limitations and considerations

This study has four key limitations and considerations to note.

To begin, facility type differed for many facilities when using a custom mapping (i.e., using the facility name since all facilities in DHIMS have the facility type within the facility name) as compared to the mapping within DHIMS. Thus, results may change with a more accurate mapping. However, it is not expected that results will substantially change (i.e., within five percentage points). All facilities were mapped to a district and region based on the export shared from DHIMS.

Second, as CD of ITNs had started before 2016, additional data were shared from 2014 and 2015. However, the activity identified substantial data quality issues at both ANC and CWC reporting during these years, including an issue where data on ITN issuance to pregnant women at ANC were reported in both the ANC and CWC data collection and reporting forms. These issues made it difficult to analyze the results with confidence. Ultimately, it was decided not to include these two years of data. Although the analyses do not display the initial two years of data shared, the results more accurately portray the true issuing rates at ANC and CWC since the quality of the data are high (i.e., between 2016 and 2021, the reporting rate, pulled from DHIMS, ranged between 91.7% and 97.0% for the ANC Monthly Form A and between 97.0% and 100.0% for the Monthly Vaccination Report), especially without any potential overlap in reporting between ANC and CWC.

Thirdly, this activity did not aim to investigate drivers to success or underperformance of facility-based ITN distribution. Routine data at facility-level was used to calculate and monitor the ITN issuing rates, which provided descriptive information on whether intended clients at facility-based health services received an ITN. Examining factors that may influence ITN issuing rates (such as facility stockouts, staffing availability, health worker knowledge and attitudes) was out of the scope of this investigation.

Lastly, there were multiple monthly anomalies in the collected data for ANC and CWC, including data with high issuing rates above 100%. Some of these anomalies are correct. For example, it is possible for a facility to have an ITN issuing rate above 100% since the denominator uses the first ANC and a pregnant women may correctly receive a net at their second ANC visit if they had not received a net already (which would cause that individual to be counted in the numerator in the month of the second visit and counted in the denominator in the month of the first visit). However, there were instances where rates reached unexpected (potentially impossible) numbers. For example, there were three instances in 2021 where a facility reported an ITN issuing rate of over 10,000%, and these are highly likely human data entry issues (e.g., in one case, the number of first ANC visits was 21 and the number of ITNs issued at ANC was 2,121, a likely instance where the data entrant entered 21 twice for the ITN value). As these anomalies could potentially be explained by the denominators used to calculate issuing rates, they were not removed from the data set. Defining anomaly as an issuing rate of greater than or equal to 500%, there were 171 instances for ANC and 281 instances for CWC in the 2016–2021 time period. This accounts to 0.06% (ANC) and 0.07% (CWC) of all data points in the time period.

## Conclusion

This activity is the first of its kind to showcase ITN issuing results to pregnant women at ANC and children under five years of age at vaccination visits at this level of detail, across multiple years of data, and to attempt to show potential factors influencing success at meeting the national strategic target of 80%. Ghana has seen improvement in ITN issuance to pregnant women and children under five at health facilities through ANC and CWC over the years. Although there are substantial differences at the regional and district levels, the country saw an overall ITN issuance of over 90% at both ANC and CWC in 2021. Considering that 97% of pregnant women received care at an ANC in the five-year range preceding Ghana’s 2019 MIS [11], it is likely that CD of ITNs through health facilities contributed to household ITN ownership, as well as targeting the most vulnerable populations.

Performance varied between the different health facility and ownership types, so it is critical to investigate what may be causing these variances in performance. Policies that limit how one type of facility functions (e.g., provision of certain services, ability to order certain commodities, inclusion in logistics or supervision systems) may play a role. Specifically, additional investigation into why publicly owned facilities tend to have higher issuing rates through ANC while privately owned facilities tend to fare better at CWC may yield recommendations and best practices that can be incorporated across all health facilities.

While it is clear that ITN distribution in Ghana has improved considerably since 2016, additional challenges surrounding ITN use as a malaria prevention tool still remain [19]. Additional work on the facilitators and barriers to ITN use in Ghana would provide valuable insights into the impact of ITNs after distribution.

However, these types of analysis, even aggregate national-level results, are currently not monitored at a global level, even within the WHO World Malaria Report. These results should therefore encourage other countries to conduct and publicize similar analyses so the global community can better understand the current situation of facility-based ITN distribution and go further to share the successes and challenges of routine distribution of ITNs through health facilities. In addition, this activity encourages additional research to be conducted to better understand influencers to highly functional distribution at health facilities. Better understanding these nuances can support more tailored trainings, policy change, and supervision of different types of facilities based on what are found to be common challenges and gaps for those different facilities. Additional research can also include investigation in the practices that succeed to maintain performance above 90% at districts and facilities and what practices or policies may impede performance or cause districts and facilities to fall in terms of performance. Other research could conduct these sorts of analysis at a more granular level, potentially within a region or within a district, looking at how facilities themselves change over time and showcase learnings based on those granular results.

## Supplementary Information

**Additional file 1: Figure S1.** Monthly issuing rate (ANC) by region. **Figure S2.** Monthly issuing rate (CWC) by region.** Figure S3.** Monthly issuing rate (ANC) by facility type. **Figure S4.** Monthly issuing rate (CWC) by facility type. **Figure S5.** Monthly issuing rate (ANC) by ownership. **Figure S6.** Monthly issuing rate (CWC) by ownership. **Figure S7.** Monthly issuing rate (ANC) by ecological zone. **Figure S8**. Monthly issuing rate (CWC) by ecological zone.

## Data Availability

Datasets can be shared upon request due to privacy results of the facility-level data.

## Abbreviations

ANC: Antenatal Care
CD: Continuous Distribution
CHAG: Christian Health Association of Ghana
CHPS: Community Based Health Planning and Services
CWC: Child Welfare Clinics
DFID: Department for International Development
DHS: Demographic Health Survey
DHIS2: District Health Information Software 2
DHIMS: District Health Information Management System
EPI: Enhanced Program for Immunization
ITN: Insecticide-Treated Bed Net
MIS: Malaria Indicator Survey
MR2: Second Dose of the Measles-Rubella Vaccine
NMEP: National Malaria Elimination Programme
PMI: President’s Malaria Initiative
UNICEF: United Nations Children’s Fund
USAID: United States Agency for International Development
WHO: World Health Organization
