# Supplementary material for: Successful implementation of ITN distribution through health facilities in Ghana
Source: Malar J. 2023 Aug 2;22:224. doi: 10.1186/s12936-023-04592-5 (PMC10398947; doi:10.1186/s12936-023-04592-5)
Supplement: Supplementary file 1 — Additional file 1: Figure S1. Monthly issuing rate (ANC) by region. Figure S2. Monthly issuing rate (CWC) by region. Figure S3. Monthly issuing rate (ANC) by facility type. Figure S4. Monthly issuing rate (CWC) by facility type. Figure S5. Monthly issuing rate (ANC) by ownership. Figure S6. Monthly issuing rate (CWC) by ownership. Figure S7. Monthly issuing rate (ANC) by ecological zone. Figure S8. Monthly issuing rate (CWC) by ecological zone. [file 12936_2023_4592_MOESM1_ESM.docx]

**Supplemental Materials**

*Figure S1. Monthly issuing rate (ANC) by region*

*Figure S2. Monthly issuing rate (CWC) by region*

*Figure S3. Monthly issuing rate (ANC) by facility type*

*Figure S4. Monthly issuing rate (CWC) by facility type*

*Figure S5. Monthly issuing rate (ANC) by ownership*

*Figure S6. Monthly issuing rate (CWC) by ownership*

*Figure S7. Monthly issuing rate (ANC) by ecological zone*

*Figure S8. Monthly issuing rate (CWC) by ecological zone*
